# Synthesis and Bioactivity of Secondary Metabolites from Marine Sponges Containing Dibrominated Indolic Systems

**DOI:** 10.3390/molecules17056083

**Published:** 2012-05-21

**Authors:** Adriano Mollica, Marcello Locatelli, Azzurra Stefanucci, Francesco Pinnen

**Affiliations:** 1 Via dei Vestini 31, Department of Pharmaceutical Sciences, Faculty of Pharmacy, University “G. d’ Annunzio” of Chieti-Pescara, Chieti 66100, Italy; 2 Piazzale Aldo Moro 5, Department of Organic Chemistry, Faculty of Chemistry, University of Rome “La Sapienza”, Roma 00197, Italy

**Keywords:** dibrominated indole, marine secondary metabolites, anticancer, natural products, alkaloids

## Abstract

Marine sponges. (e.g., *Hyrtios* sp., *Dragmacidin* sp., *Aglophenia pleuma*, *Aplidium cyaneum*, *Aplidium meridianum*.) produce bioactive secondary metabolites involved in their defence mechanisms. Recently it was demonstrated that several of those compounds show a large variety of biological activities against different human diseases with possible applications in medicinal chemistry and in pharmaceutical fields, especially related to the new drug development process. Researchers have focused their attention principally on secondary metabolites with anti-cancer and cytotoxic activities. A common target for these molecules is the cytoskeleton, which has a central role in cellular proliferation, motility, and profusion involved in the metastatic process associate with tumors. In particular, many substances containing brominated indolic rings such as 5,6-dibromotryptamine, 5,6-dibromo-*N*-methyltryptamine, 5,6-dibromo-*N*-methyltryptophan (dibromoabrine), 5,6-dibromo-*N,N*-dimethyltryptamine and 5,6-dibromo-L-hypaphorine isolated from different marine sources, have shown anti-cancer activity, as well as antibiotic and anti-inflammatory properties. Considering the structural correlation between endogenous monoamine serotonin with marine indolic alkaloids 5,6-dibromoabrine and 5,6-dibromotryptamine, a potential use of some dibrominated indolic metabolites in the treatment of depression-related pathologies has also been hypothesized. Due to the potential applications in the treatment of various diseases and the increasing demand of these compounds for biological assays and the difficult of their isolation from marine sources, we report in this review a series of recent syntheses of marine dibrominated indole-containing products.

## 1. Introduction

The marine world consists of a large variety of ecosystems covering a wide range of temperatures, nutrients, and lighted/unlighted zones. Due to this large variability, organism diversification ranges from planktonic organisms to mammals. Thirty years ago, interest in marine organisms started to develop: in fact, bioactive secondary metabolites from marine sponges involved in their defence mechanisms may have possible application in medicinal chemistry, representing a valid starting point for new drug development processes [1]. More than 5,000 products from sponges and related microorganisms are studied, and over 200 new metabolites from these natural sources are reported each year [2]. Recently a review [3] reported that over 1,000 new compounds were isolated only in 2009, together with relevant biological activities. At the same time the tools for the isolation, purification and structural elucidation had improved, especially related to novel instrument configurations such as hyphenated techniques (e.g., HPLC-MS/MS and HPLC-NMR) [4,5]. Researchers have focused their attention principally on the metabolites with anticancer and cytotoxic activity. There are many biological targets for anticancer drugs; between them the cytoskeleton has a crucial role in the cellular proliferation, motility and profusion involved in the metastatic process associated with tumours. Many substances containing a brominated indolic ring isolated from different marine sources have shown anti-tumoural activity, antibiotic and anti-inflammatory properties. Due to the potential application in the treatment of various diseases, the growing demand for these compounds for *in vitro* and *in vivo* pharmacological studies and the low natural concentration generally related to these secondary metabolites, the development of synthetic approaches is essential. In this review, we report a summary of dibrominated indole-containing compounds extracted and isolated from marine sources and the chemical pathways used for their synthesis.

### 1.1. Metabolites from Hyrtios sp.

**Figure 1:**
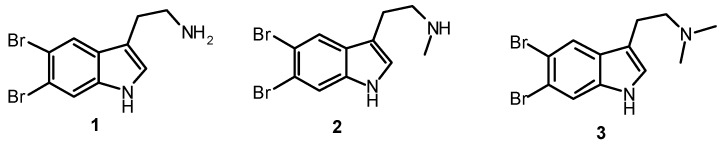

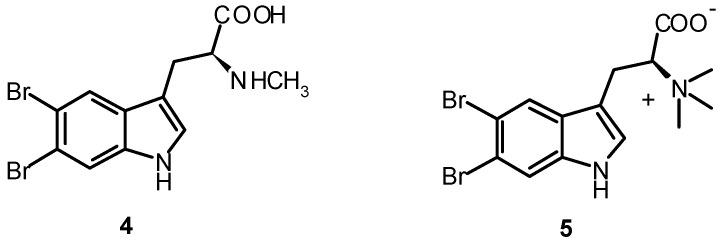
Chemical structures of isolated antibacterial metabolites from *Hyrtios* sp.

The antibacterial metabolites 5,6-dibromotryptamine (**1**), 5,6-dibromo-*N*-methyl-tryptamine (**2**), 5,6-dibromo-*N,N*-dimethyltryptamine (**3**), 5,6-dibromo-*N*-methyltryptophan (dibromoabrine) (**4**), and 5,6-di-bromohypaphorine (**5**) have been isolated from *Hyrtios* sp. found in the South Pacific area [6] (Figure 1).

These products, were isolated by Reversed Phase High Performance Liquid Chromatography (RP-HPLC) on octadecylsilane (ODS) stationary phase, and fully characterized by NMR, High Resolution Mass Spectrometry (HR-MS), and specific optical rotatory power (only for compound **5**) and they been tested as bee venom PLA2 inhibitors. Compounds **3** and **5** (IC_50_ = 0.2 mM) showed a highly activity as antioxidants in the qualitative DPPH test quantified by the ORAC assay (Table 1).

Compounds **1** and **4** show moderate cytotoxic activity toward the HCT-116 colon carcinoma cell line and toward p53 cell lines [7]. Considering the structural analogy between the endogenous monoamine serotonin and the marine indolic alkaloids, a possible use of some dibrominated indolic metabolites in the treatment of depression-related pathologieshas been hypothesized; the activity of **3** has been evaluated with the forced swimming test on mice and with the anxiety/depression test in chickens [8] in which it showed a significative anti-depressive activity at 20 mg/Kg and anxiolitic activity at 30 mg/Kg [9].

### 1.2. Metabolites from Dragmacidin sp.

**Figure 2:**
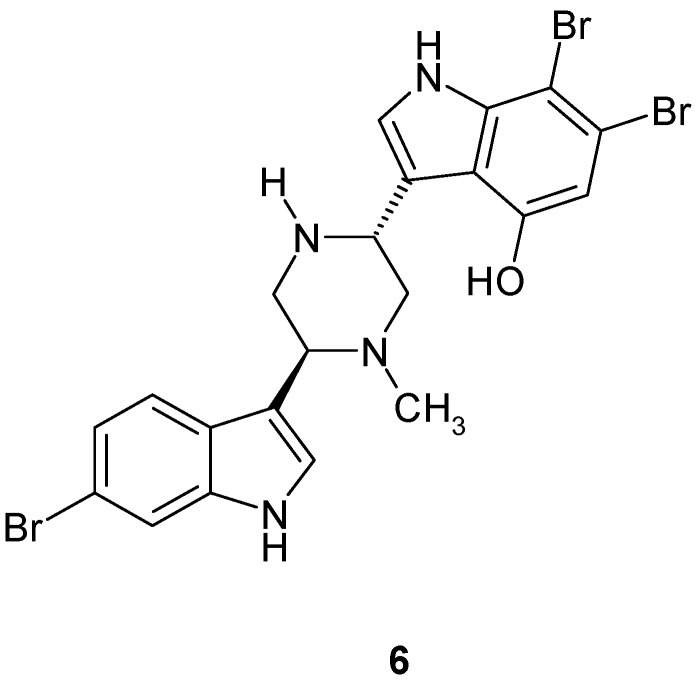
Structure of dragmacidin isolated from *Dragmacidin* sp.

Dragmacidin (**6**, Figure 2), whose skeleton is characterized by a piperazinic ring condensed to two substituted indole rings, was isolated from *Dragmacidin* sp. Dragmacidin inhibits leukemic murine cells P388 (IC_50_ = 15 μg/mL), the A-549 lung cells, HCT-8 (colon) and MDMB (breast cancer) with an IC_50_ = 1–10 μg/mL *in vivo*. Recent data also demonstrated a high inhibition activity for bee venom PLA2 [10].

### 1.3. Metabolites from Aglophenia pleuma

**Figure 3:**
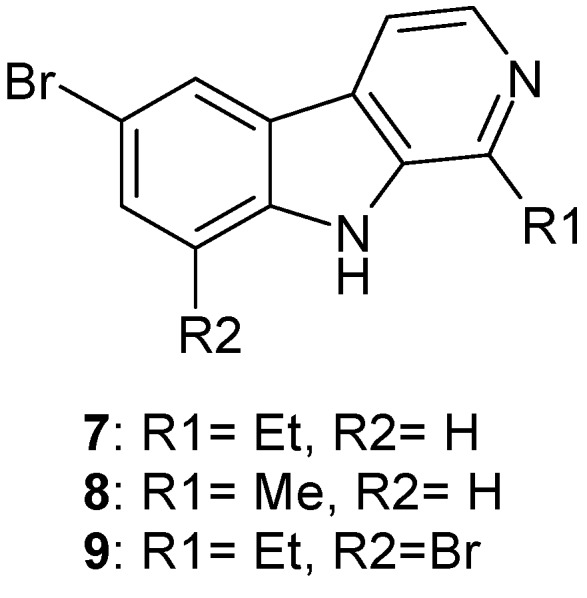
Brominated-β-carboline structures isolated from *Aglophenia pleuma*.

The brominated-β-carbolines **7**–**9** (Figure 3) were isolated from *Aglophenia pleuma* and characterized. Their activity is based on the effect exerted on the genic expression of the endogenous protein VEGF involved in inducible hypoxia; brominated-β-carbolines are involved in the inhibition of angiogenesis in growing solid tumours [11].

### 1.4. Metabolites from Aplidium meridianum

**Figure 4:**
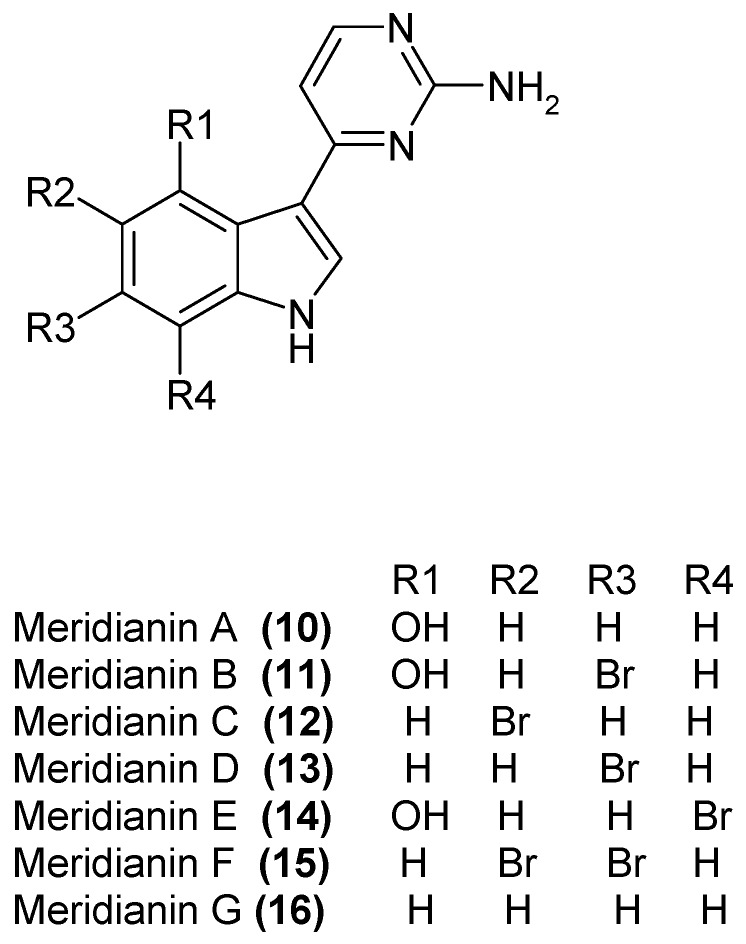
Meridianin structures isolated from *Aplidium meridianum*.

In 1998, the groups of Walker, Hernandez and Palermo reported, for the first time, the isolation of five 3-(2-aminopyrimidine)-indoles, namely meridianins A–E (**10**–**14**, Figure 4), from the tunicate *Aplidium meridianum* collected at a depth of 100 m near the South Georgia Islands (South Atlantic) [12,13]. The structures were deduced by 2D-NMR spectroscopy and comparison to literature compounds. The re-examination of the crude extracts using tandem mass spectrometry (MS/MS) lead to the discovery of two new meridianins, compounds F (**15**) and G (**16**) [13] (Figure 4).

From the original isolation studies of the meridianins, was known that the members of the meridianin family had cytotoxic properties [14]. Meijer and co-workers discovered that the meridianin family inhibits several protein kinases, including CDK1, GSK3, and protein kinase A42 and investigated the origin of these properties. The variety of naturally occurring meridianins allowed to a small investigation of structure-activity relationships. The unsubstituted meridianin skeleton found in meridianin G (**16**) was only weakly inhibitory, while a single bromine for hydrogen substitution at the 5- or 6- position of the indole ring [meridianin C (**12**) and D (**13**), respectively] resulted in considerable improvements in potency, with up to 1000-fold decreases in IC_50_ observed in favorable cases.

Interestingly, in meridianin F (**15**), the presence of two bromine atoms, at both the 5- and 6-positions, resulted in improved potency relative to meridianin G (**16**), but decreased potency compared to either monobrominated meridianins C (**12**) or D (**13**). Substitution of a hydroxyl group for hydrogen at the indole 4-position, as seen in meridianin A (**10**), resulted in smaller improvements in potency, while a bromine at position 7 and a hydroxyl group at the 4-position [as seen in meridianin E (**14**)] resulted in the most potent compounds, with IC_50_ values in the low M to high nM range, depending on the target.

### 1.5. Metabolites from Aplidium cyaneum

**Figure 5:**
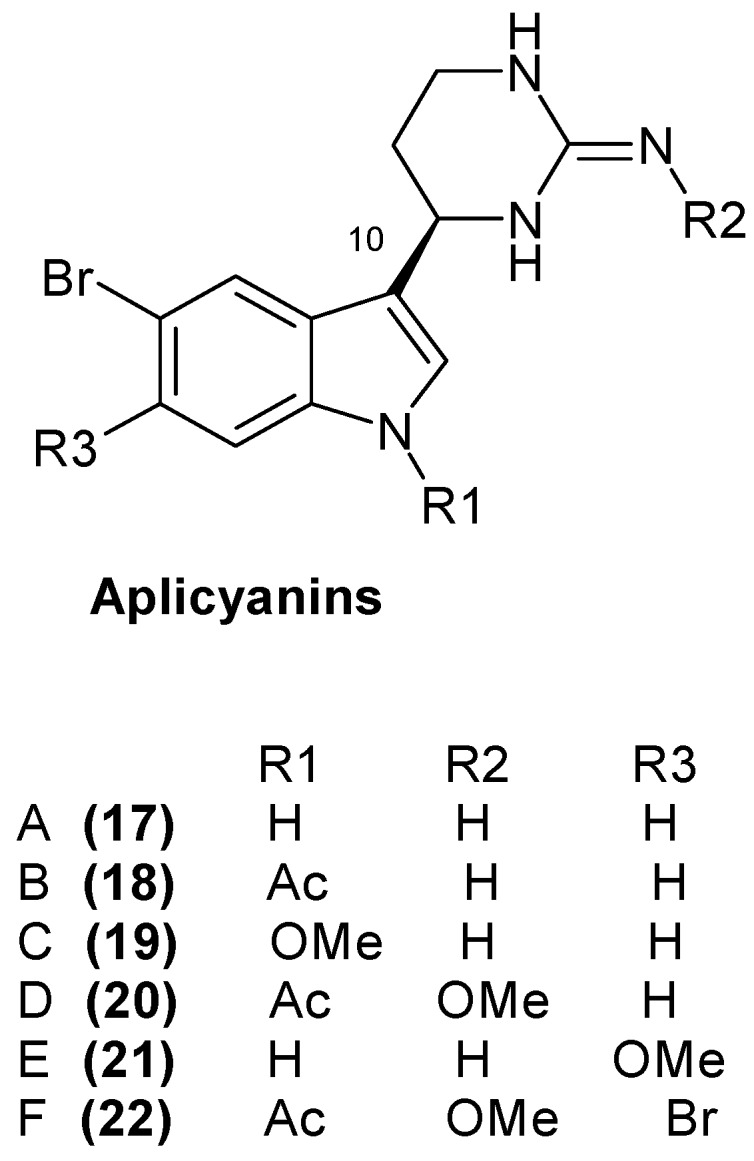
Aplicyanin structures isolated from the tunicate *Aplidium cyaneum*.

Recently, an additional series of compounds with a similar structure to the meridianins was discovered. The aplicyanin family was isolated from the Antarctic tunicate *Aplidium cyaneum* and consists of six variants **17**–**22** on a core 3-(tetrahydropyrimidyl)-indole structure [15] (Figure 5). In the aplicyanin family, the indole ring is further modified by the addition of bromine substituents and, in several members, by the addition of an unusual *N*-methoxy group. Unlike the planar pyrimidine ring of the meridianins, the tetrahydropyrimidine system of the aplicyanins has a stereocenter at *C*-10. Optical rotation measurements indicate that this stereocenter is non-racemic in the aplicyanins, and analysis of isolated aplicyanin E (**21**) by chiral high performance liquid chromatography (HPLC) indicates the natural product was isolated as a single enantiomer. However, the absolute configuration of this center remains undetermined.

Aplicyanins B (**18**), D (**20**), and F (**22**) have been found to have significant cytotoxic and antimitotic activities, with IC_50_ values in the low to sub-*μ*M range. Aplicyanins A (**17**) and C (**19**) were found to possess no cytotoxic or antimitotic activity at the concentrations tested, while aplicyanin E (**21**) possessed weak cytotoxic activity.

These results indicate a vital role for the acetyl portion on the guanidine group [12]. Two biological activities were evaluated in parallel during the fractionation of the tunicate extracts: cytotoxic activity, was evaluated against a panel of three human tumor cell lines, including colon (HT-29), lung (A-549), and breast (MDA-MB-231) [15], and antimitotic activity, which was measured using a specific microplate immunoassay (ELISA). Both activities were evaluated for all six of the compounds isolated. Cytotoxic activity in the sub-μM range as well as antimitotic properties were found for compounds **18**, **20**, and **22**, whereas compounds **17** and **21** proved to be inactive at the highest concentrations tested and compound **17** displayed only mild cytotoxic properties. These results clearly suggest a key role for the presence of the acetyl group at *N*-16 in the biological activity of this family of compounds. Aplicyanin E posses only a moderate cytotoxic activity, contradictory statements showing the importance of the acetyl group on the guanidine moiety [14].

### 1.6. Metabolites from Pycnoclavella kottae

**Figure 6:**
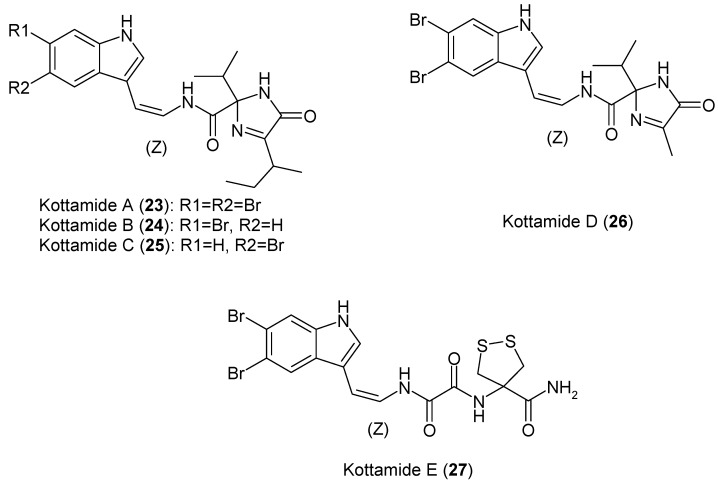
Kottamide structures isolated from *Pycnoclavella kottae*.

From the ascidian *Pycnoclavella kottae*, found in the proximity of Three Kings Islands in New Zeland, kottamides A–E (compounds **23**–**27**) have been isolated (Figure 6) [16]. Kottamides represent the first natural compounds containing a disubstituted indole portion and a 1,2-dithiolancarboxylic acid residue (Adt), which is a synthetic derivative of cysteine [17,18]. Kottamide D (**26**) was investigated for anti-inflammatory and anti-metabolic properties in microplate assays using the cell-impermeable tetrazolium salt 2-(4-iodophenyl)-3-(4-nitrophenyl)-5-(2,4-disulfophenyl)-2*H-*tetrazolium monosodium salt(WST-1) and the intermediate electron acceptor 1-methoxyphenazine-methosulfate (PMS) [19].

Compound **26** exhibited potent anti-metabolic activity (IC_50_ 6–10 μM) with both anti-proliferative and anti-inflammatory activity in the 2–200 μM range. In a short-term metabolic assay over 1.25 h, **26** inhibited the reduction of WST-1 by human HL60 and Jurkat cells by 83–92% at 40 μM, while over 48 h, **26** inhibited proliferation of HL60 cells by 66% at 20 μM and 9% at 2 μM. Similar results were obtained with the non-transformed interleukin-3-dependent murine cell line, 32Dcl23. Under the same conditions, the tubulin-stabilizing anti-cancer drug taxol inhibited the reduction of WST-1 by only 3–5% in the short-term assay but after 48 h it inhibited proliferative responses by 64–81% at 1 μM. In an anti-inflammatory assay using activated human peripheral blood neutrophils, **26** inhibited superoxide production generated in response to the inflammation promoting agents *N*-formylmethionyl leucyl-phenylalanine (fMLP) and phorbol myristate acetate (PMA) in the range 95–100% (200 μM concentration) [20]. In contrast, taxol did not inhibit the fMLP response and only weakly inhibited PMA-induced superoxide production. Kottamides A–D (**23**–**26**) were also assayed for a range of cytotoxic and antimicrobial properties. All four kottamides exhibited moderate P388 activity with IC_50_ values of 20, 14, and 36 μM for compounds **23**, **24**/**25** mixtures, and **26**, respectively. Further evaluation of **23** at the NCI revealed only modest cytotoxic activity. Compound **23** was also tested for cytotoxic/antiviral activity against the African Green Monkey kidney cell line (BSC-1) infected with the RNA virus PV110 and was found to have moderate cytotoxic activity and some antiviral activity. Compound **23** exhibited no antimicrobial activity against the bacteria *Bacillus subtilis* and *Escherichia coli* or the fungi *Candida albicans* and *Trichophyton mentagrophytes* in disk assays. A plausible biogenesis of **23**–**25** involves stereospecific imidazolone ring formation from modified Trp-Val-Ile and Trp-Ile-Ala tripeptide precursors. Even if no biological activity has been reported for kottamide E (**27**), its unique structure makes it an interesting synthetic target in organic chemistry.

## 2. Synthesis of Dibrominated Indole-Containing Derivatives

**Scheme 1:**
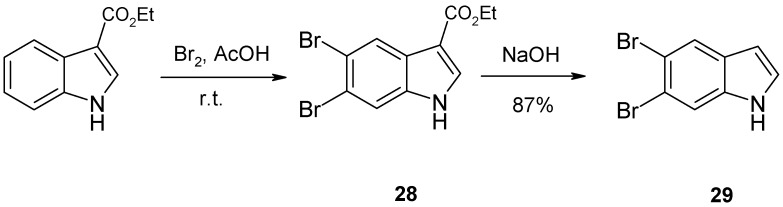
Selective bromination starting from ethyl indole-3-carboxylate by Majima and co-workers [21].

Due to the extreme importance of biological substances containing dibrominated indolic rings, their insufficient amounts present in their marine organism sources and the difficulties in isolation, there is a great need for total or partial synthesis of those products. The actual synthetic methods of halo-substituted indoles present some difficulties: (i) long reaction time; (ii) difficulties to obtain the starting materials; (iii) the use of extremely toxic reagents; (iv) significant side reactions; (v) expensive catalysts; (vi) the lack of selectivity in substitution reactions on the indole ring. A first approach to the synthesis of the monobrominated indole ring was proposed early by Majima and co-workers in 1930, starting from ethyl indole-3-carboxylate to give 5,6-dibromo-indole (**28**) in high yield [21].The regioselectivity of this reaction was determined by a 3-step degradation of **28** to 3,4-dibromoaniline, and comparison of melting points with an authentic sample (Scheme 1). Analytical data for **29** was unsurprisingly sparse given available techniques of the period, with only microanalysis being reported.

**Scheme 2:**
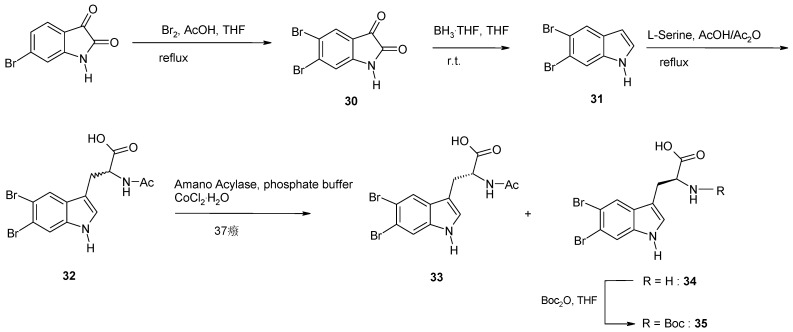
Synthesis of dibromotryptophan derivatives by Mollica and co-workers [22].

Recently, our research group reported the synthesis of dibromotryptophan derivatives starting from the commercially available 6-bromoisatin [22]. The synthetic protocol is reported in Scheme 2. After the selective procedure of bromination described by Vine and co-workers [23], 5,6-dibromoisatin (**30**) was carefully purified by repeated recrystallization from AcOH. Then the α-keto-amide group was reduced by a solution of BH_3_ in THF to give 5,6-dibromoindole (**31**) in good yield (68%). As previously reported, attempts to obtain **31** by using LiAlH_4_ or NaBH_4_ were unsuccessful, while reduction with LiBH_4_ in THF gave only very low yields [24]. Synthesis of the 5,6-dibromotryptophan derivative **32** was performed by exploiting the nucleophilic reactivity of the indole ring at the C-3 carbon atom. Thus, 5,6-dibromoindole (**31**) was reacted with (*S*)-serine in a mixture of acetic acid/acetic anhydride to give the desired *N*-acetyl-(*R*,*S*)-5,6-dibromotryptophan (**32**) in good yield. The reaction proceeds through *N*-acetyl-α,β-unsaturated species as electrophilic intermediates leading, as expected, to the complete loss of the (*S*)-serine chiral centre. “Amano acylase”, an enzyme commonly used for optical resolutions of racemic *N*-acetyl amino acids was used to resolve the racemic *N*-acetyl derivative **32**. As previously reported the two large substituents at the 5- and 6-positions of the indole ring sensibly slow down the enzymatic hydrolysis rate as compared with the *N*-acetyl-(*R,S*)-tryptophan. Thus, in order to obtain in one step the (*S*)-form as derivative suitable for peptide synthesis, the usually reported procedures [25], have been modified.

**Scheme 3:**
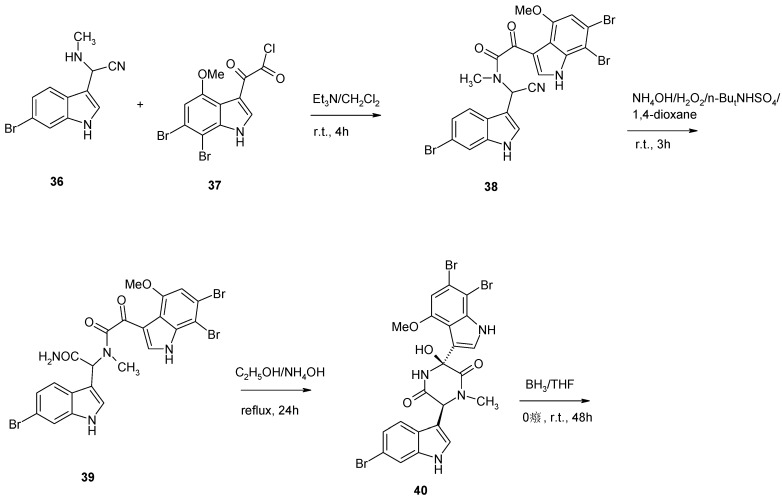

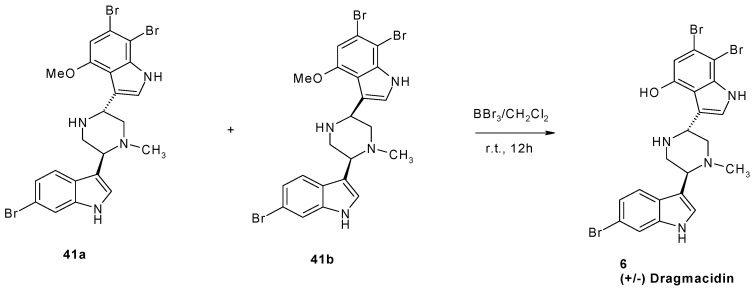
(+/−) Dragmacidin synthesis reported by Jiang and co-workers [10].

The aqueous buffered solution was acidified (pH 3) and extracted with EtOAc to give *N*-acetyl-(*R*)-5,6-dibromotryptophan (**33**) in ca. 90% enantiomeric excess (e.e.). Subsequent evaporation of the aqueous layer and treatment of the residue with *tert*-butyl dicarbonate gave the desired enantiopure *N*-Boc-(*S*)-5,6-dibromotryptophan (**35**), ca. 82% e.e. The e.e. of products **33** and **35** was tested by chiral HPLC by following the method reported by Jin and co-workers [26]. In 1994 Jiang and co-workers reported the first synthesis of racemic dragmacidin **6** [10] (Scheme 3).

*N*-methylaminonitrile **36** was reacted directly with acid chloride **37** to yield stable *N*-methylamide **38** in 92% yield. The next key intermediate, piperazinedione **40**, was obtained by hydrolysis of the nitrile with ammonium hydroxide/hydrogen peroxide in the presence of a phase transfer catalyst followed by intramolecular cyclization of the resulting ketoamide **39** in refluxing alcohol containing 30% aqueous ammonium hydroxide. The cyclization evolved cleanly, and compound **40** was isolated as the sole product. Reduction of **40** with borane-tetrahydrofuran at 0 °C afforded a mixture of *o*-methyl-dragmacidins **41a** and **41b** in a ratio of 4:1. It was apparent from the magnitude of the coupling constants observed for the H2 and H5 methylene protons in the piperazine ring of **41a** that the piperazine ring was in a chair conformation with both indolyl substituents in an equatorial orientation. On the other hand, the coupling constants of H2 and H5 in **41b** show that the 6-bromoindolyl is in equatorial orientation and the 6,7-dibromoindolyl is in an axial orientation. The product ratio in this reduction was very dependent on the reaction temperature; reduction at room temperature gave a 1:l mixture of **41a** and **41b**. This selectivity can be explained by elimination of the C2 hydroxyl group prior to 1,4-reduction. Finally, to complete the synthesis, **41a** was *O*-demethylated with boron tribromide, smoothly affording racemic dragmacidin. The ^1^H-NMR chemical shifts and coupling constants of synthetic dragmacidin (**6**) correspond to those reported for dragmacidin isolated from nature. 

**Scheme 4:**
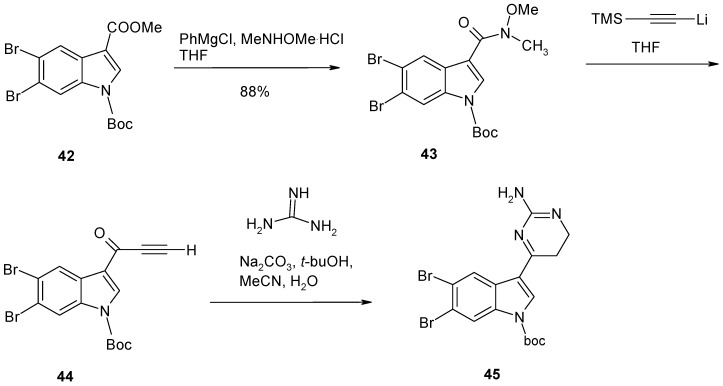
Meridianin F synthesis reported by Palermo and co-workers [13].

In 2007, Palermo and co-workers proposed a concise synthesis of meridianine F (**45**) in only five steps [13] (Scheme 4). The meridianins are marine natural products that have attracted some interest from the scientific community due to their promising biological activity, including protein kinase inhibition and anticancer activity [27]. However, a synthesis of meridianin F has not been previously reported, presumably because of the inaccessibility of the 5,6-dibromoindole substitution pattern. A concise, 5-step synthesis of meridianin F (**45**) [28], containing the characteristic 2-aminopyrimidine ring at C-3 common to all the meridianins, is shown in Scheme 4. The *N-*Boc-carbamate **42** was converted into the Weinreb amide **43**. Treatment of **43** with lithium(trimethylsilyl)acetylide gave the terminal alkyne **44** directly. Direct conversion of the alkynyl ketone **44** to meridianin F (**45**) was achieved using the conditions of Muller, developed for the synthesis of related meridianins and analogues. This methodology, which occurs with concomitant Boc deprotection, was previously reported on TMS-protected acetylenes and extended to alkyl and aryl substituted alkynes, with reaction times of 38 h at 80 °C, or more generally 120 °C overnight.

The shorter reaction time in the case of **44** (2 h, 80 °C) is presumably due to the use of the non-TMS protected compound. Analytical data for **45** were in agreement with those previously reported, and the route shown in Scheme 4 therefore represents the first synthesis of meridianin F.

**Scheme 5:**
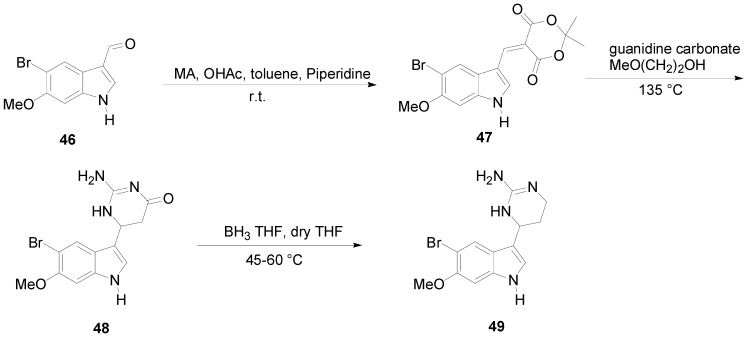
Aplicyanin E synthesis reported by Síša and co-workers [28].

In 2009, Síša and co-workers proposed the synthesis of aplicyanins A, B, E, F and other 14 analogues [28] (Scheme 5).

To decrease the electronic density of the conjugated double bond a malonic ester derivative such as Meldrum’s acid (MA) was used (Scheme 5) [29]. Thus, the Meldrum acid-indole adduct **47** was prepared in good yield by following the procedure described by Benzies and co-workers [30]. Reaction between adduct **47** and guanidine carbonate in refluxing 2-methoxyethanol gave the 2-amino- dihydropyrimidone **48** in yields that varied according to the indole substituent. The *N*-methoxy-derivative **48** was only obtained in 20% yield. The poor results for the *N*-methoxy derivatives can be rationalized by two factors. The electron donating character of the methoxy in position 1 of the indole diminishes the reactivity of compound **47** toward nucleophilic addition of guanidine. Thus, these derivatives are relatively non-reactive and, consequently, require longer times to consume the starting material in the guanidine addition-cyclization reaction. Second, they confer instability and low solubility to the 2-aminodihydropyrimidin-4-one **48**. Reduction of compound **48** with BH_3_-THF [31] afforded the aminotetrahydropyrimidine **49** in good yield. The reduction conditions for compound **48** had to be strictly controlled because longer reaction times or higher temperatures led to the loss of the *N*-methoxy group. Reduction of **48** produced **49** in 30% yield.

**Scheme 6:**
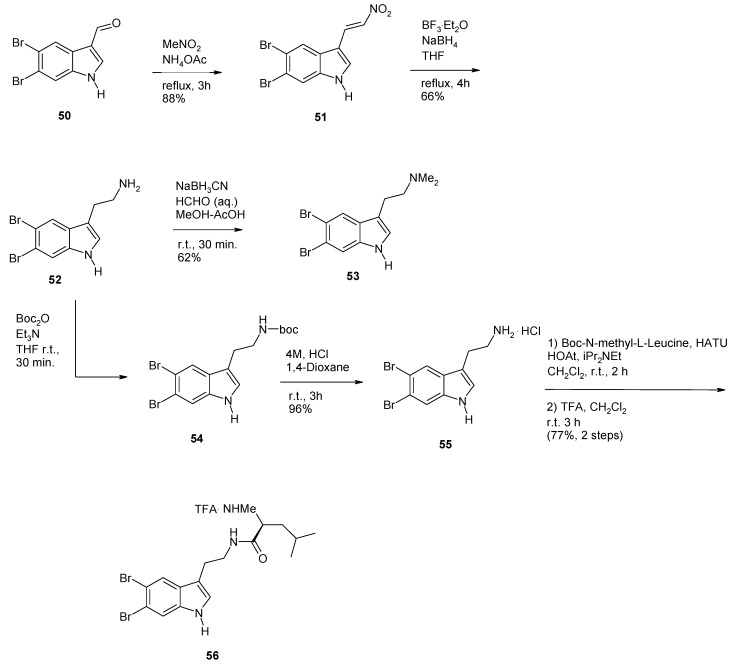
Alternatamide D (**56**) synthesis reported by Sperry and co-workers [32].

Recently the synthesis of alternatamide D (**56**) a peptidic antibiotic isolated from *Amanthia alternata* and the two intermediate products 5,6-dibromotryptamine (**52**) and 5,6-dibromo-*N,N*-dimethyltryptamine (**53**) (Scheme 6) has been reported [32].

With higher quantities (>1 g) of 5,6-dibromoindole-3-carbaldehyde (**50**) readily available, the synthesis of 5,6-dibromotryptamine (**52**) was undertaken. The Henry reaction of **50** in nitromethane at reflux in the presence of ammonium acetate proceeded smoothly, furnishing the novel nitroalkene **51**. Initially, the key reduction of **51** to the desired 5,6-dibromotryptamine (**52**) gave disappointing results, with lithium aluminium hydride [33] and various hydrogenation conditions [34] resulting in extensive debromination of the indole ring. However, employing *in situ* generated BH_3_ in THF [35] gratifyingly effected nitroalkene reduction with no detriment to the dibrominated indole, and affording 5,6-dibromotryptamine (**52**) in good yield.

Spectroscopic data of (**52**) were in good agreement with the literature values. 5,6-dibromo-tryptamine (**52**) was subjected to reductive dialkylation affording 5,6-dibromo-*N,N*-dimethyl-tryptamine (**53**), a natural product with significant antidepressant properties [9]. The spectroscopic data of (**53**) matched those of the natural product [36]. Due to the small amount of impurities present and its tendency to undergo degradation after an extended period, 5,6-dibromotryptamine (**52**) was converted into its *tert*-butyloxycarbonyl (Boc) derivative **54** and subjected to purification by flash chromatography. The Boc group was then removed from **54** with anhydrous HCl in 1,4-dioxane, giving the hydrochloride salt of 5,6-dibromotryptamine that is stable for months at room temperature (Scheme 6). Unfortunately, **55** failed to undergo amide coupling with *N*-methyl-L-leucine despite attempts using a plethora of peptide coupling agents.

This result was not surprising as there is a dearth of examples reporting *N*-methylleucine as a willing participant in amide couplings presumably due to the unprotected secondary amine present in it. This problem was solved by subjecting **55** and Boc-*N*-methyl-L-leucine to HATU and HOAt in the presence of Hünig’s base, giving Boc-alternatamide D that was immediately deprotected with trifluoroacetic acid (TFA) in dichloromethane, affording alternatamide D (**56**, Scheme 6). The amide coupling was presumed to proceed without racemisation as uronium-based coupling agent was employed [37].

## 3. Conclusions

Natural products have been for centuries a rich source of valuable agents in medicine [38]. More than half of currently available drugs [39] are natural compounds or are related to them, and in the case of cancer this proportion surpasses 60%. Additionally, many new natural compounds of diverse structures, isolated from plant sources [40,41] and mammals have been considered prototypes, leads or heads of series and their later structural modification have afforded compounds with pharmacological activity and extraordinary therapeutic possibilities, e.g., [42,43,44,45,46,47,48]. Pharmacomodulation is one of the methods used to search for new drugs and consists in taking as the “lead compound” a chemical substance of established structure and of known biological activity; for example, camptothecin [44] is a lead natural anticancer drug which structural modification has led to more potent and less toxic compounds than the prototype. Otherwise, the possibility of generating hybrids of natural products [49] seems to be very promising in the development of new compounds with better activity than that of the parent compound. In this review we reported few examples of recent syntheses of marine dibrominated indole containing products: the bromination of tryptophan and its derivatives appears to occur widely in marine organisms, especially in sponges, tunicates and algae. These brominated compounds arise through a broad spectrum of post-translational modification enzymes, e.g., bromo- and lacto-peroxidases and are likely to be defensive compounds, endowed with strong pharmacological activity; therefore intensive research is still needed on isolation and characterization of dibromo-indole-containing peptidic and non-peptidic compounds. Today, technical drawbacks associated with natural products have been lessened, and there are better opportunities to explore the biological activity of previously inaccessible sources of natural products [50]. In the light of the fact that chemical diversity of natural products is well suited to provide the core scaffolds for future drugs, there will be further developments in the use of novel natural products of marine origin and chemical libraries based on natural products, in drug discovery process. 

## Figures and Tables

**Table 1:** PLA and ORAC values for cited compounds (**1**, **2**, **3**, **4** and **5**).

Compound	PLA_2_^a^	ORAC _FL_^b^
5,6-Dibromotryptamine (**1**)	0.62 ± 0.01	nt
*N*-Methyl-5,6-dibromotryptamine (**2**)	0.33 ± 0.03	nt
*N,N*-Dimethyl-5,6-dibromotryptamine (**3**)	0.77 ± 0.05	0.06 ± 0.01
5,6-Dibromoabrine (**4**)	0.30 ± 0.01	0.07 ± 0.01
5,6-Dibromo-L-hypaphorine (**5**)	0.20 ± 0.01	0.22 ± 0.04

**^a^** IC_50_ values (mM ± SEM; n = 2) on bee venom PLA_2_. Manoalide (positive control) IC_50_ 0.5 ± 0.05 M; **^b^** ORAC values are expressed as relative Trolox equivalent. Fluorescein (FL). Relative ORAC value = [AUC product − AUC blank)/(AUC Trolox − AUC Blank)] × (molarity Trolox/molarity product) (n = 3). Molarity in mM. AUC: Area Under the Curve. AUC blank = AUC obtained for the second control FL + AAPH. Ascorbic acid (positive control) ORAC _FL_ 0.95 ± 0.02; nt = not tested.
